# Providers’ Perceptions of Respectful Maternity Care and Enabling Conditions in a Regional Hospital: A Qualitative Study

**DOI:** 10.3390/ijerph22101570

**Published:** 2025-10-15

**Authors:** Sthembile P. Zwane, Lawrence Chauke

**Affiliations:** 1 Department of Obstetrics and Gynaecology, School of Clinical Medicine, Faculty of Health Sciences, University of the Witwatersrand, Witwatersrand, Johannesburg 2000, South Africa; zwanepr@gmail.com; 2 Department of Obstetrics and Gynaecology, Charlotte Maxeke Johannesburg Academic Hospital, Johannesburg 2193, South Africa

**Keywords:** provider, perceptions, respectful, maternity care

## Abstract

Globally, women continue to die from pregnancy-related conditions that could be prevented through ensuring timely access to emergency obstetric care and facility-based deliveries supervised by skilled birth attendants. However, many women are reluctant to deliver in maternity healthcare facilities due to the widespread disrespect and abuse that patients have reportedly received. Respectful maternity care has been identified amongst the possible solutions. This study explored perceptions of respectful maternity care and the enabling conditions of a multidisciplinary group of maternity healthcare providers working at a busy, specialised public mother and child regional hospital in Gauteng, South Africa. An explorative, descriptive, and contextual study design with a phenomenological perspective was adopted. Semi-structured interviews were conducted with each of the 30 purposefully selected study participants. The interviews were digitally recorded, professionally transcribed, and analysed using Tesch’s Constant Comparison method. Two main categories, namely (1) healthcare providers’ perceptions of respectful maternity care and (2) enabling conditions for its practice emerged, encompassing seven themes: women-centred care, provision of high-quality care, preservation and promotion of women’s rights, creating an enabling environment for the practice of RMC, in-service training, accountability of healthcare providers for their actions, and community involvement. The perceptions of the study participants regarding respectful maternity care align with global standards; however, successful implementation requires the establishment of enabling conditions.

## 1. Background

Globally, women continue to die from preventable pregnancy-related conditions that could be prevented through timely access to emergency obstetric services and delivering in healthcare facilities under the supervision of skilled birth attendants [1,2]. However, women are reluctant to deliver in maternity healthcare facilities due to the widespread disrespect and abuse that patients have reportedly receive. Respectful maternity care (RMC) has been proposed as one possible solution to this problem. Women who receive RMC are likely to feel safe, trust their caregivers, and, as a result, deliver in healthcare facilities with the help of skilled birth attendants [3]. Delivering in healthcare facilities would provide better access to emergency care, resulting in an increased number of positive pregnancy outcomes [2].

Between 2000 and 2017, maternal deaths declined by 38% globally (World Health Organisation) [1]. This decline translates to a reduction of 2.7% per day, a figure which is far below the 6.4% reduction required per day to achieve global commitment to the United Nation’ Sustainable Development Goal (SDG) to reduce the maternal mortality ratio to 70/100,000 livebirths or less by 2030 [1,2,3]. Furthermore, compared to high-income countries (HICs), low- and middle-income countries (LMICs) continue to bear the highest burden of maternal mortality (99% of the global burden, 66% of which occurs in Sub-Saharan Africa) [1]. Even more disturbing is the fact that the majority of maternal deaths could be prevented if they have timely access to emergency obstetric (EMOC) care and delivery in maternity healthcare facilities under the supervision of skilled birth attendants [2]. This approach has been associated with a reduction in maternal and neonatal deaths [1,2,3,4]. However, some women avoid delivering in maternity healthcare services because of fear of being abused and mistreated [5,6,7].

RMC is one of the strategies that has been proposed to improve quality of care and encourage women to deliver in healthcare facilities where they can receive appropriate care [6,7]. The execution of high-quality care standards requires not only functional healthcare systems, indispensable knowledge, and expertise from healthcare providers but also a welcoming and supportive maternity care environment [8,9]. Therefore, in 2016, the WHO incorporated RMC into the maternity healthcare services quality framework [9]. The framework is composed of eight domains of quality—spanning both provision and experience of care and specific standards and measurable quality statements for each domain. This approach aims to reduce preventable mortality and morbidity by ensuring that every woman and newborn receives high-quality, respectful, and evidence-based care.

South Africa, a medium-industrialised country, also encounters multiple hardships in the healthcare sector. With close to a million births each year, maternity care providers are faced with high workload and sporadic availability of resources. The healthcare system is further overstretched, with demands from escalating numbers of illegal foreign nationals migrating into the South African healthcare system, as well as enormous numbers of South Africans seeking care in public healthcare facilities; these are some of the factors known to increase abuse, disrespect, and substandard care in maternity healthcare facilities [10,11,12,13]. The same studies also documented the existence of widespread abuse and disrespect in maternity units in South Africa. This abuse occurs in environment where pregnant women are supposed to receive safe, quality and respectful maternity care, consequently undermining their trust on the health system meant to provide safe and respectful maternity care, undermining trust in the health system and, the country’s efforts to achieve meaningful reduction in maternal mortality. Addressing this challenge requires systemic reforms, policy enforcement, training of maternity healthcare providers on RMC and, accountability mechanisms to promote respectful, rights-based care.

This study explored the perceptions of respectful maternity care and its enabling condition among a group of a multiprofessional team of maternity healthcare providers working at a busy, specialised public mother and child academic hospital in Johannesburg, Gauteng Province, South Africa. While the topic of RMC has been widely explored, less attention has been paid on the perceptions of multidisciplinary team of maternity healthcare working in public healthcare maternity units with a high number of deliveries. Exploring perceptions of RMC among multiprofessional teams of maternity healthcare providers working in busy public maternity units is crucial to uncover differences in values, practices, and systemic pressures that shape patient–provider interactions. This insight could assist in designing targeted interventions that foster collaborative, compassionate care and reduce disrespect and abuse across all cadres of maternity healthcare providers.

## 2. Methods

Because the focus of this study was on the perceptions of maternity healthcare providers working in a specific setting, we decided to perform an explorative, descriptive, and contextual study that utilised phenomenological perspective as a measuring instrument.

### 2.1. Study Setting

This study was conducted at a specialised public mother and child academic regional hospital in Johannesburg, Gauteng Province, South Africa. This location is regarded as one of the busiest regional hospitals in Johannesburg, delivering, on average, the children of 15,000 women per annum. The hospital is located in a historically disadvantaged area and the population serviced by this facility is wholly dependent on government services. Furthermore, the study site has been on the news and was the subject of Ombudsman investigation following allegations that included pregnant women sleeping on the floor because of overcrowding shortage of both medical and nursing staff, and infrastructure-related challenges that include constant water related challenges [14].

### 2.2. Study Population

The target population comprises midwives, operational managers, obstetrics registrars (residents), and consultants who were working at the study site during the study period.

### 2.3. Study Sample and Sampling Technique

The study sample comprises healthcare providers supervising and rendering direct maternity services at the study site (including those who had been rotating in other academic hospitals as part of residency training) who had been providing maternity care for a minimum of two years. The study participants were selected using the following inclusion criteria: experienced maternity care providers including unit manager/supervisor, nurse, or doctor with two or more years of consistently working in the maternity unit. These criteria ensured that the study participants were experienced maternity healthcare providers. Purposive sampling was used as a sampling strategy, and sample size through saturation (no new information emerging from the interviews).

### 2.4. Data Collection

Data collection took place from the 1st of October to the 31st of December 2021 using semi-structured interviews with individual participant maternity healthcare provider. All the interviews were conducted by the principal investigator. A series of open-ended questions from an interview guide were used to elicit the required information. Participants were interviewed at a time and place that was convenient for them. Only the English language was used as it is the language of communication at the study site. Verbal communication strategies such as probing, paraphrasing, and reflecting on the participants were used to encourage participants to elaborate on their views. Each interview lasted between 25 and 60 min; the interviews were digitally recorded and professionally transcribed. In addition to the interviews, field notes were written to capture researcher’s observations and reflections of the interviews.

### 2.5. Data Management and Availability

All digital copies of the interview transcripts and field notes were stored in a password-protected personal computer. The data is available from the principal investigator.

### 2.6. Data Analysis

Data analysis began with bracketing, which refers to the researcher’s removal of all preconceived expectations about the phenomenon under investigation [15]. Data analysis followed Tesch’s eight-step approach [16]. This approach involves reading through the transcripts to make sense of the data, analysing the transcriptions, listing and assigning codes to topics generated, and thereafter assembling and re-coding the data to create themes and categories.

### 2.7. Trustworthiness

This study’s trustworthiness was ensured with the use of four quality criteria (credibility, transferability, dependability, and confirmability), discussed in detail below [15,16,17,18,19].

Credibility or internal validity refers to the truthfulness of the study findings. Truthfulness of the perceptions of healthcare providers was ensured through prolonged engagement, triangulation, and peer debriefing. Each interview lasted between 25 and 60 min, and the interviews were conducted in a natural setting; that is, at the study site. The researcher used a variety of data sources, i.e., interviews, observations, and field notes, to ensure data triangulation. The researcher further made use of an independent coder to co-code the transcribed data and ensure that the analysis was a true reflection of the participants’ perceptions and to provide new perspectives on the analysis.

Nowell et al. [17] describe transferability as the extent to which the findings of a study are confirmed by or applicable to a different group in a different setting. To ensure the transferability of this study, a solid description of the study setting, research methodology, and participants’ demographics has been provided, along with supporting verbatim quotes from the participants. This approach would ensure transferability of the methodology of the study even when the actual findings might differ from one setting to another.

Dependability refers to how the research findings are consistent and sufficiently accurate to establish the trustworthiness of the study. A pilot study was conducted with two participants before the main study to enhance consistency, but the results of the pilot data were not included in the main study. Furthermore, the researcher collected data until data saturation was achieved; this refers to the point at which the interviews did not provide new insights into the phenomenon under investigation.

Lastly, confirmability (objectivity) refers to the neutrality of the study findings in terms of accurately reflecting information and the inquiry and not being a product of the researcher’s biases and prejudices. This criterion was achieved using member checks whereby the researcher gave the completed study report to five of the participants to read and verify its truthfulness. From these member checks, some necessary amendments were made to the findings.

However, the authors were consistently aware of the potential impact of their personal assumptions and beliefs on the processes of data collection, analysis, and interpretation of results. The authors are both a midwife (also serving as the Maternity Unit Manager) and an Obstetrician and Gynaecologist (also Clinical Head of the Department) at a quaternary hospital in the same region. As a result, the authors have maintained a heightened awareness of how their experiences and knowledge of the subject may influence the research outcomes. To address this, we have employed reflective practices to minimise the introduction of personal biases into the study and to enhance the trustworthiness of the findings.

### 2.8. Ethical Considerations

Permission to conduct this study was obtained from the Chief Executive Officer at the study site, and ethics clearance was provided by the Human Research Committee (HREC) of the University of the Witwatersrand (reference number: M210408, 23 August 2021). All participants provided informed written consent for the interviews and digital recordings prior to their participation.

## 3. Results

### 3.1. Description of the Sample

A total of 30 participants took part in the study (Table 1). Seven themes that can be grouped into two categories—providers’ perceptions of RMC (three themes) and enabling conditions for practice of RMC (four themes)—emerged (Table 2).

### 3.2. Category 1: Providers’ Perceptions of RMC

Most of the study participants had a positive outlook regarding RMC. They saw RMC as a tool that could (1) assist in building rapport between patients and maternity healthcare providers; (2) promote cooperation between patients and healthcare workers; (3) improve patient outcomes, including reducing maternal deaths; (4) provide support to patients with anxiety and emotional and social problems; and (5) assist providers with delivering quality maternity care and adhering to the standards of maternity practice.

The above was captured by the themes that emerged from the providers’ perceptions of RMC, i.e., women-centred care, provision of high-quality care, and preservation and promotion of women’s rights.

#### 3.2.1. Theme 1: Women-Centred Care

The theme women-centred care included four subthemes, namely holistic care, multidisciplinary approach, allowing for companionship or support during labour, empathy and support. These are discussed in detail below.

##### Subtheme 1: Holistic Care

The healthcare providers believed that RMC should include cultural, psychosocial, and spiritual support in addition to addressing women’s medical needs:

It is not just the medical management from an obstetrics perspective but also bringing in the psychological and emotional as well as socio-cultural support that a pregnant woman needs.

(41-year-old, Consultant, 16 years of experience in maternity care)

##### Subtheme 2: Multidisciplinary Approach

Healthcare providers were of the view that RMC can only be achieved by multidisciplinary teams with patient participation instead of individuals/a single professional group, such as midwives, alone:

RMC is all about being aware and acknowledging that there are role-players involved in our daily duties as maternity service providers. Being a trained professional does not mean I know and can solely accomplish everything. Most importantly my patients also need to give their view on what I do for them or to them.

(52-year-old, Operational manager, 30 years of experience in maternity care)

##### Subtheme 3: Allowing for Companionship/Support During Labour

Some providers identified allowing the mother to have a companion in labour as an important component of RMC:

So, I think most of our negative experiences and outcomes that we get in our unit can be easily corrected by a simple respectful approach, allowing women to be with their preferred companions during labour and always being there with and for labouring women. Besides. “Midwife” simply means being with a woman.

(35-year-old, midwife specialist, 12 years of experience in maternity care)

##### Subtheme 4: Respect, Empathy, and Quality of Care

Respect, empathy, and provision of high-quality care were also identified as essential components of RMC.

Me, as an individual, if I was in a patient’s shoes, I would really want to be treated with respect and given proper care regardless of who I am or where I come from, so it makes sense, and it is appropriate that we do the same for our patients.

(31-year-old, midwife specialist, 9 years of experience in maternity care)

Respectful maternity care is all about empathy and quality care. It is about treating patients with respect, gentleness, and then communicating with them. I think those are the most compassionate things really. A lot can happen so over and again you need to reassure the moms by communicating with them, how far they are dilated, what is the next thing to do etc.”

(26-year-old, midwife, 4 years of experience in maternity care)

#### 3.2.2. Theme 2: Provision of High-Quality Care

The provision of high-quality care had three aspects: individualised care, adherence to professional practice and professionalism, and practicing evidence-based care.

##### Subtheme 1: Individualised Care and Respect for Cultural Beliefs and Practices

According to healthcare providers, RMC includes individualised care, which must take into consideration a woman’s cultural beliefs and practices.

I think that it is very important to give respect and treat pregnant women as human beings with specific needs, just like you and me. And they should also be respected in their cultural beliefs and practices.

(31-year-old, basic midwife, 5 years of experience in maternity care)

##### Subtheme 2: Adherence to Professional Practice and Professionalism

Adherence to professional principles and standards emerged several times during the interviews of both participant obstetricians and midwives.

Respectful maternity care involves respecting your profession, as we are professional midwives, we are governed by the SANC. We need to respectfully adhere to the stipulated regulations governing us and it also goes to respecting the patients and their rights that are stipulated in the Constitution of our country, as well as just respecting the patient as a human being. So, RMC is considering and in cooperating all those aspects in our daily duties.

(35-year-old, midwife specialist, 14 years of experience in maternity care)

##### Subtheme 3: Practicing Evidence-Based Care: Avoiding Unnecessary Interventions

Evidence-based (scientifically proven) maternity care was viewed as an important component of RMC.

So, we have a lot of old myths and practices, in both medicine and nursing and this is something we need to stop. Unfortunately, youngsters coming into the healthcare profession copy these and regard them as normal and acceptable. I think continuous teaching of both our older and younger population, within and beyond the maternity field, on what is scientifically proven is the way to go.

(38-year-old, consultant, 14 years of experience in maternity care)

#### 3.2.3. Theme 3: Preservation and Promotion of Women’s Rights

The third theme on providers’ understanding of RMC was preservation and promotion of women’s rights. Ethical and dignified care and respecting women’s cultural rights emerged from this theme.

##### Subtheme 1: Ethical and Dignified Care

Three ethical principles (autonomy, non-maleficence, and beneficence) were linked with RMC:

Respectful maternity care is affording childbearing women the opportunity or a safe measure in which they can seek health care when they need to, and respectfully by abiding by their autonomy, offering them non-maleficence and beneficence in terms of always making sure that whatever you provide to them is in their best interest.

(48-year-old, registrar, 12 years of experience in maternity care). (27-year-old, basic midwife, 7 years of experience in maternity care)

Well, I can say though that respectful care is not and should not be only clinical. Doing a vacuum-assisted delivery or doing a caesarean section is all clinical and it’s taken from the protocol book, but the protocol book doesn’t teach you how to incorporate women’s socio-cultural and spiritual dimensions in your care. The healthcare worker, the patients, and other people such as families are 41 supposed to work collaboratively to produce healthy mothers, babies and ultimately a healthy nation.

(39-year-old, consultant, 15 years of experience in maternity care)

##### Subtheme 2: Respecting Women’s Rights

According to the healthcare provider, respect for women’s rights goes beyond physical care; it also includes psychosocial, spiritual, and cultural rights:

My understanding of respectful maternity care is that it is the dignified approach or treatment that is being given or rendered to pregnant women, which promotes include cooperation between the woman and her caregivers and include cooperation of the whole charter of human rights. A whole in sense that this is not confined to respecting their physical rights only but also their psychosocial, spiritual, and cultural rights.

(41-year-old, Consultant, 16 years of experience in maternity care)

### 3.3. Category 2: Enabling Conditions for the Practice of Respectful Maternity Care

This category outlines the enabling conditions required for the expression and practice of RMC.

#### 3.3.1. Theme 1: Creation of an Enabling Environment for Healthcare Providers

There were three aspects to creating *an enabling environment for healthcare providers*, namely supervisory support and involvement, provision of a safe working environment and employee wellness programmes, and availability and equitable distribution of healthcare resources.

##### Subtheme 1: Supervisory Support and Involvement

All participant maternity healthcare providers stated that visibility of managers, supportive visits, and motivation from managers have a direct impact on service delivery, including the practice of RMC. In addition, providers pointed out that another issue that has a strong hold on whether RMC is practiced or disregarded is the availability of trusted confidential employee assistance programmes aimed at assisting employees in dealing with both non-work and work-related challenges.

Managers, play a huge role towards the care we give for example, negative or non-constructive feedback from our managers creates unnecessary pressure on us and sometimes we feel intimidated. Working under such pressure or situation is not good at all for our patients as we end up projecting our frustrations towards them.

(30-year-old, basic midwife, 5 years of experience in maternity care)

##### Subtheme 2: Provision of a Safe Working Environment and Employee Wellness Programmes

A safe working environment and supporting employee wellness were identified to be among the enabling conditions for the practice of RMC.

It is my recommendation that we get some form of wellness programmes which somehow allow people to express themselves freely when it comes to their professional and social issues—the workplace should be the safe environment for people to express their issues and get them sorted out if there is a way to. They say, “behind a healthy mother and baby is a happy midwife”.

(56-year-old, midwife specialist, 33 years of experience in maternity care)

##### Subtheme 3: Availability and Equitable Distribution of Healthcare Resources

Most providers mentioned that the management of human and material resources impacts the practice of RMC and service delivery in general.

This hospital has an overflow of patients, and we don’t have as much space, staff, and equipment to accommodate large numbers all the time. So, I think we need urgent procurement of staff, stock, and equipment because it is frustrating and tiring for doctors and midwives to be unable to render the expected care with and witness unpleasant patient care outcomes because of close-to-zero resources. How is then a frustrated employee expected to deliver respectful care?

(26-year-old, basic midwife, 4 years of experience in maternity care)

#### 3.3.2. Theme 2: In-Service Education

All participant providers emphasised in-service training as an important enabler of practicing RMC.

I think in-service training programmes covering burning topics like RMC for both clinical staff and managers should be in place in all maternity care institutions because you would find that most people might know about such, but they don’t actually understand it. The more people know about concepts such as RMC through regular talks and teachings, the greater the chances of practicing it.

(53-year-old, operational manager, 20 years of experience in maternity care)

#### 3.3.3. Theme 3: Accountability: Holding Healthcare Workers Responsible for Their Actions

Holding healthcare providers accountable for their actions was also identified as an enabler of RMC.

The core of our daily duties as midwives is to provide equal, supportive, and non-humiliating treatment to women presenting at our facilities regardless of their social, cultural, and religious background. Failure for healthcare providers to practice the above should be a disciplinable offense. Incidents of disrespect and disciplinary measures should not be treated as “under the carpet” matters so that all maternity service providers can learn that there is no room for disrespect in our facilities.

(35-year-old, registrar, 12 years of experience in maternity care)

#### 3.3.4. Theme 4: Community Education and Involvement

Most providers considered education and community involvement in childbearing issues and safe practices are critical for the success of the implementation of RMC including its practice.

So, communication and education are major keys, I can say that most of our maternity care problems are related to lack of information in the communities. The public needs to be informed of the different levels of care and services available at each level. For example, a woman who has had a previous\caesarean section delivery needs to know that she cannot be assisted in an MOU when in labour.

(44-year-old, basic midwife, 18 years of experience in maternity care)

I mean if we look at childbirth at the time of our great grandmothers, delivering babies was a community effort and so the holistic approach was there. You know certain cultures like in my own culture your mom, your mom in law and all other women experienced in childbirth processes and practices would come together to assist—you would deliver, you would have, a special type of bath and rituals to ensure that you and your baby are safe and healthy.”

(35-year-old, midwife specialist, 10 years of experience in maternity care)

## 4. Discussion

Studies, including those from South Africa [11,12,13] have raised concerns regarding the lack of respectful and empathetic care in some of the country’s maternity units, including instances of verbal abuse and neglect during childbirth. These forms of abuse encompass cultural insensitivity and a disregard for women’s privacy, resulting in emotional trauma and leaving women feeling powerless and unheard. Conversely, according to these studies, positive experiences were associated with compassionate staff, clear communication, and treatment with dignity. Additionally, these studies also reported that systemic issues such as understaffing, inadequate training, and weak accountability structures, contribute to the reported abuses. This qualitative study examined the perceptions of a multiprofessional team of maternity healthcare providers at a busy, specialised public Mother and Child Regional Academic Hospital in Johannesburg, Gauteng Province, South Africa, concerning respectful maternity care and enabling conditions that facilitate its practice.

The study sample comprised a diverse group of maternity healthcare providers, including nurses with basic midwifery training, specialised midwives, trainee specialists in obstetrics, and qualified obstetricians and gynaecologists. Participants’ ages ranged from 25 to over 55 years, with experience varying from 2 to more than 20 years. The diversity and experience of the study sample brough richness to the study. Overall, the perceptions of RMC by the study participants were positive, providing insight into what they believed RMC was about, including enabling conditions for its practice. They perceived RMC as an important and critical component of the provision and quality of maternity healthcare services. Their perception of RMC focused on three key areas: (1) women-centred care, (2) provision of high-quality care, and (3) preservation and promotion of women’s rights. Furthermore, healthcare providers understood women-centred care to be a holistic (takes into consideration physical, emotional, spiritual, cultural factors/needs) care approach that is delivered by a multidisciplinary team in an empathetic and supportive manner. This support is provided by both healthcare providers and labour companions. The second theme, provision of high-quality care, focuses on the provision of maternity healthcare that is individualised, based on available evidence and best practices, avoids unnecessary interventions, adheres to professional practice, and is delivered in a professional manner. The third and final theme in this category uses a human rights framework and refers to healthcare providers’ obligation to preserve and promote women’s rights, which involves, but is not limited to, always acting in an ethical and dignified manner and respecting women’s cultural rights.

The participants’ perceptions of RMC reported above are congruent with both the international literature [20,21,22,23,24] and local studies [11,12,13,25] on the topic and are therefore encouraging. These perceptions are in keeping with the WHO’s ‘Standards for quality maternal and newborn care in health facilities, WHO Framework for Intrapartum Care and the framework for elimination of disrespect and abuse during pregnancy and childbirth [3,9,26]. Like our study, a study conducted in Rwanda, reported that healthcare workers had positive attitude towards RMC [27]. Providers participating in this study perceived RMC as encompassing the delivery of women-centred and high-quality maternity care, alongside the preservation and promotion of women’s rights. Similar findings were reported by Moridi et al. in a study conducted in Iran [22]. Similarly to our study, this Iranian study had subthemes that included protecting women’s dignity, supporting them in labour, and creating a safe labour environment, including involving women in decision-making. These findings perhaps suggest that the perceptions of RMC are similar across different cultures and settings.

Furthermore, the study participants included the provision of high-quality maternity care in their perceptions of RMC. Quality of care referred to both technical (technical competence of providers and evidence-based care) and emotional (women’s experience of care) aspects. These elements of care are also part of the WHO Standards for Improving Quality of Maternal and Newborn Care in Health Facilities [9]. The study participants underscored that the provision of substandard care services constitutes a form of disrespect and can discourage women from seeking maternity care in the future. Similar findings have been reported by other studies [10,11,12,13,20,21,22,23,24,25,27]. These studies highlighted the negative impact of poor-quality maternity care—often attributable to a lack of resources, inadequately skilled birth attendants, and poor working conditions for healthcare providers—on women’s experiences of care and health outcomes. Women who experience poor quality care during pregnancy may be compelled to seek care elsewhere in future pregnancies.

An additional significant finding from this study is the perception that RMC encompasses the preservation and promotion of women’s rights. Study participants characterised RMC as the provision of consensual care that integrates ethical and legal principles into the daily practice of maternity care. This approach acknowledges and demonstrates and understanding that women originate from diverse socio-cultural backgrounds and the roles these background play in their decision-making processes. The human rights-based approach to RMC is further supported by the Respectful Maternity Care Charter developed by the White Ribbon Alliance [28]. This charter clarifies and vividly articulates the importance of respecting the rights of women and newborns while receiving maternity care within healthcare facilities. These are rights to freedom from harm and ill-treatment, confidentiality and privacy, dignity and respect, equality, freedom from discrimination and equitable care, timely and highest attainable level of healthcare, liberty, autonomy, self-determination, freedom from oppression, access to information, informed consent, and respect for personal choices and preferences, including companionship during maternity care.

Furthermore, the participants in this study strongly expressed the importance of creating conditions that enable RMC to be successful. The foremost enabling condition was the creation of a supportive environment for maternity healthcare service providers. They believed that the establishment and maintenance of an enabling and supportive environment strongly lie with the management teams of maternity healthcare facilities and hospitals. They further expressed that such an environment can be accomplished through supervisors’ or managers’ visibility, involvement, and support in clinical areas, as well as the availability and fair distribution of the resources necessary for rendering safe maternity healthcare services. Similar views were reported in a study conducted in Limpopo, South Africa [25]. The midwives in the Limpopo study expressed the view that they found it extremely difficult to deliver safe and quality care to maternity patients because of the stressful working environment, lack of support, and unconstructive criticism from management if something in the maternity wards went wrong. Similar findings were also reported by Ndwiga et al. in their study conducted in Kenya [24].

Mothiba et al. further alluded to the existence of system-wide barriers that are believed to be responsible for poor-quality care, let alone a lack of respectful maternity care [25]. This important finding highlights the importance that health system factors play in the provision of RMC. Creating enabling conditions for RMC also features in the seminal work performed by Shakibazadeh et al. on RMC [21]. In addition to the above, in-service training emerged during the interviews as a vital and enabling driver to the implementation and practice of RMC. Participants also raised the need for awareness and regular staff training on not just clinical care guidelines but also the non-clinical aspects of care, such as respectful maternity care, including the impact of incorporating or disregarding such care aspects on patients’ experiences of care and health outcomes. A study performed by Rosen et al. in 2015 identified deficits in maternity health workers’ knowledge to be among the anti-drivers of RMC highlight the need for skill development amongst the possible solutions [20]. These authors further highlighted the existing gaps in the education of professionals, the prevalence of medicalized rather than woman-centred care, the lack of respectful maternity care (RMC) in the undergraduate training of health teams, suboptimal behavioural practices that do not emphasise respectful care or evidence-based approaches, the scarcity of training sites that can serve as models for the provision of RMC in educational institutions, as well as the absence of in-service education or training for continuous staff updates on best practices. These factors are among the key drivers of disrespect and abuse in maternity care.

Additionally, participants in this study indicated that the successful implementation of the RMC framework within healthcare necessitates holding maternity healthcare providers and their supervisors accountable for their actions. It is widely recognised that accountability promotes vigilance and diligence in professional conduct. Furthermore, healthcare providers have expressed strong opinions on accountability, asserting that instances of disrespect and abuse towards childbearing women should lead to significant disciplinary measures. Afulani and Moyer emphasised the necessity for countability in the provision of maternity care in their commentary published in The Lancet in 2019 [29].

Lastly, our study participants affirmed that childbearing issues extend beyond healthcare facilities to encompass communities and the public at large. Ongoing engagement and integration of maternity services that align with the values of various communities are essential strategies for achieving positive maternity care outcomes. This perspective is supported by Rosen et al., who emphasise that the implementation of community activities, including educational campaigns, is a vital approach to addressing the issue of respectful care [20].

## 5. Strengths and Limitations

This study was carried out in a single mother and child academic regional hospital in Gauteng. While the results obtained may not reflect the perceptions of maternity healthcare providers on RMC in regional hospitals in other areas as well as across different levels of care, this study provides valuable insights regarding the perceptions of RMC of maternity healthcare providers who work in busy environments. A larger study including different levels of care in different geographical regions was preferable, but this was not possible due to time limitations and the national COVID-19 regulations. This study only explored healthcare providers’ perceptions of RMC and did not explore pregnant women’s perceptions or their experiences. A study exploring both healthcare providers’ and women’s perspectives and experiences of RMC with an observational element to verify these findings would have provided insights into not only providers’ theoretical knowledge of RMC but also actual practices. This approach would have assisted in identifying areas that needed attention to improve women’s experiences of maternity care and health outcomes. Regardless of these limitations, this study adds to the growing body of knowledge on RMC and supports the WHO’s effort to make RMC standard practice in all units providing maternity healthcare services globally.

## 6. Conclusions

According to the best of the researchers’ knowledge, this qualitative study is the first study to explore the perceptions of respectful maternity care and the enabling conditions by a multiprofessional team of maternity healthcare providers working in a busy, specialised public mother and child hospital in a low-resource setting. The maternity healthcare providers’ perceptions of RMC and enabling conditions for its practice identified in this study are in keeping with the published literature, suggesting that perceptions of RMC are not bound by context or culture. This study suggests that maternity healthcare workers are well-versed in the principles of RMC, but they require enabling conditions in order to successfully practice RMC. The findings of this study have significant implications for maternity policy and practice in South Africa and other low- and middle-income countries, particularly those grappling with ongoing resource challenges and overcrowding in public hospitals. Additionally, these findings are pertinent to the training of maternity healthcare providers and the reform of health systems. Therefore, we strongly encourage all key stakeholders to foster an environment that supports the implementation of Respectful Maternity Care and to incorporate RMC into the training curricula for healthcare students and maternity care providers.

## Figures and Tables

**Table 1:** Demographic characteristics of study participants.

Characteristic (n = 30)	Category	Actual Number	Percentage
Age	25–35 years	14	46.7%
	35–45 years	11	36.75%
	45–55 years	4	13.3%
	>55 years	1	3.3%
Gender	Female	17	56.7%
	Male	13	43.3%
Qualification/Position	Basic midwives/accoucheurs	7	23.3%
	Midwife specialists	8	26.8%
	Operational managers	4	13.3%
	Registrars	7	23.3%
	Consultants	4	13.3%
Years of Experience	<2 years	0	0%
	2–10 years	15	50.0%
	10–20 years	8	26.7%
	20–30 years	6	20.0%
	>30 years	1	3.3%

**Table 2:** Categories, main themes, and subthemes derived from maternity healthcare providers’ perceptions of respectful maternity care and enabling conditions for its practice.

Category	Theme	Subthemes	Number of Instances Each Theme or Subtheme Came up During Interviews (n = 30)
Providers’ perceptions of RMC	Women-centred care	Holistic care	30
		Multidisciplinary approach	28
		Allowing companionship/support during labour	18
		Respect, empathy, and quality of care	22
	Providing high-quality care	Individualised care and respect for cultural beliefs and practices	28
		Adherence to professional practice and professionalism	15
		Practicing evidence-based care: avoiding unnecessary interventions	18
	Preservation and promotion of women’s rights	Ethical and dignified care	29
		Respecting women’s cultural rights	24
Enabling conditions for the practice of RMC	Creation of an enabling environment for healthcare workers	Supervisory support and involvement	30
		Provision of a safe working environment and employee wellness programmes	30
		Availability and equitable distribution of healthcare resources	30
	In-service education		21
	Accountability: holding healthcare workers responsible for their actions		15
	Community education and involvement		20

## Data Availability

The data used in this study are included in the manuscript; anonymized data are available from the authors upon request.

## References

[1] WHO (2019). Trends in Maternal Mortality 2000 to 2017: Estimates by WHO, UNICEF, UNFPA, World Bank Group and the United Nations Population Division: Executive Summary. Appswhoint [Internet]. https://apps.who.int/iris/handle/10665/327596.

[2] Chauke L. (2025). Improving access to emergency obstetric care in low- and middle-income countries. Best Pract. Res. Clin. Obstet. Gynaecol..

[3] World Health Organisation (2018). Intrapartum Care for a Positive Childbirth Experience [Internet]. https://apps.who.int/iris/bitstream/handle/10665/260178/9789241550215-eng.pdf.

[4] Hawkes S., Buse K. (2019). Sustainable Development Goals. Public Health.

[5] Bohren M.A., Vogel J.P., Hunter E.C., Lutsiv O., Makh S.K., Souza J.P., Aguiar C., Saraiva Coneglian F., Diniz A.L., Tunçalp Ö. (2015). The Mistreatment of Women during Childbirth in Health Facilities Globally: A Mixed-Methods Systematic Review. PLoS Med..

[6] Singh K., Brodish P., Suchindran C. (2014). A Regional Multilevel Analysis: Can Skilled Birth Attendants Uniformly Decrease Neonatal Mortality?. Matern. Child Health J..

[7] Miller S., Lalonde A. (2015). The global epidemic of abuse and disrespect during childbirth: History, evidence, interventions, and FIGO’s mother−baby friendly birthing facilities initiative. Int. J. Gynecol. Obstet..

[8] Asefa A., McPake B., Langer A., Bohren M.A., Morgan A. (2020). Imagining maternity care as a complex adaptive system: Understanding health system constraints to the promotion of respectful maternity care. Sex. Reprod. Health Matters.

[9] World Health Organization (2016). Standards for Improving Quality of Maternal and Newborn Care in Health Facilities [Internet].

[10] Masibigiri T., Mudau A.G., Manyuma D. (2024). Women’s Experiences Regarding Maternity Care in a Selected Hospital in Vhembe District, Limpopo Province: A Qualitative Approach. Healthcare.

[11] Hastings-Tolsma M., Nolte A.G.W., Temane A. (2018). Birth stories from South Africa: Voices unheard. Women Birth.

[12] Malatji R., Madiba S. (2020). Disrespect and Abuse Experienced by Women during Childbirth in Midwife-Led Obstetric Units in Tshwane District, South Africa: A Qualitative Study. Int. J. Environ. Res. Public Health.

[13] Hosseini Tabar J., Shahoie R., Zaheri F., Mansori K., Hashemi Nasab L. (2023). Prevalence of disrespect and abuse during childbirth and its related factors in women hospitalized in the postpartum ward. J. Fam. Med. Prim. Care.

[14] South African Department of Health (2025). Investigation Report into Allegations Against Rahima Moosa Mother and Child Hospital (RMMCH).

[15] Fischer C.T. (2009). Bracketing in qualitative research: Conceptual and practical matters. Psychother. Res..

[16] Tesch R. (1990). Qualitative Research Analysis Types and Software Tools.

[17] Nowell L.S., Norris J.M., White D.E., Moules N.J. (2017). Thematic analysis: Striving to Meet the Trustworthiness Criteria. Int. J. Qual. Methods.

[18] Creswell J.W. (2014). Research Design: Qualitative, Quantitative, and Mixed Method Approaches.

[19] Lincoln Y., Guba E.G. (1985). Naturalistic Inquiry.

[20] Rosen H.E., Lynam P.F., Carr C., Reis V., Ricca J., Bazant E., Bartlett L.A. (2015). Direct observation of respectful maternity care in five countries: A cross-sectional study of health facilities in East and Southern Africa. BMC Pregnancy Childbirth.

[21] Shakibazadeh E., Namadian M., Bohren M., Vogel J., Rashidian A., Nogueira Pileggi V., Madeira S., Leathersich S., Tunçalp Ö., Oladapo O.T. (2017). Respectful care during childbirth in health facilities globally: A qualitative evidence synthesis. BJOG.

[22] Moridi M., Pazandeh F., Hajian S., Potrata B. (2020). Perspectives of respectful maternity care during childbirth: A qualitative study. PLoS ONE.

[23] Balde M.D., Diallo B.A., Bangoura A., Sall O., Soumah A.M., Vogel J.P., Bohren M.A. (2017). Perceptions and experiences of the mistreatment of women during childbirth in health facilities in Guinea: A qualitative study with women and service providers. Reprod. Health.

[24] Ndwiga C., Warren C.E., Ritter J., Sripad P., Abuya T. Exploring Provider Perspectives on Respectful Maternity Care in Kenya: “Work with What You Have”. https://reproductive-health-journal.biomedcentral.com/articles/10.1186/s12978-017-0364-8.

[25] Mothiba T.M., Skaal L., Berggren V. (2019). Listen to the Midwives in Limpopo Province South Africa: An Exploratory Study on Maternal Care. Open Public Health J..

[26] World Health Organization (2014). WHO Statement: The Prevention and Elimination of Disrespect and Abuse During Facility-Based Childbirth.

[27] Uwamahoro V., Semasaka J.P.S., Ndagijimana A., Humuza J. (2023). Perceptions and attitudes of midwives on respectful maternity care during childbirth: A qualitative study in three district hospitals of Kigali City of Rwanda. Pan Afr. Med. J..

[28] White Ribbon Alliance Respectful Maternity Care: The Universal Rights of Childbearing Women. www.whiteribbonalliance.org/respectfulcare.

[29] Afulani P.A., Moyer C.A. (2019). Accountability for respectful maternity care. Lancet.

